# Thrombotic Complications in Immune Thrombocytopenia Patients Treated with Avatrombopag

**DOI:** 10.3390/hematolrep15030054

**Published:** 2023-09-12

**Authors:** Mahmoud Abdelsamia, Saira Farid, Steven Dean, Spero R. Cataland

**Affiliations:** 1 Department of Medicine, Michigan State University, East Lansing, MI 48824, USA; 2 Department of Medicine, Ohio State University, Columbus, OH 43210, USA

**Keywords:** avatrombopag, venous thrombosis, ITP

## Abstract

Avatrombopag is a novel oral non-peptide thrombopoietin receptor agonist (TPO-RA) that was approved by the FDA as a second-line therapy for chronic immune thrombocytopenia (cITP). Avatrombopag has shown promising results in regards to efficacy and tolerability, but to our knowledge, there are no reports of thrombotic complications associated with avatrombopag. We present two patients with chronic ITP who suffered thromboembolic events shortly after starting treatment with avatrombopag. The first case is that of a 30-year-old female with refractory cITP who failed multiple lines of ITP therapy and was hospitalized with an intracranial bleed. The patient eventually recovered after an emergent splenectomy but subsequently developed a right lower lobe pulmonary embolism three weeks after starting treatment with avatrombopag. The second case is that of a 58-year-old female with a prolonged history of ITP, and no prior history of peripheral vascular disease, who suffered from both arterial and venous thrombotic events four weeks after starting avatrombopag. Given the new arterial and venous thrombotic complications, avatrombopag was stopped. She was challenged with avatrombopag again and developed yet another thrombotic complication.

## 1. Introduction

Avatrombopag is an orally administered medication that was approved in 2018 for the treatment of chronic immune thrombocytopenic purpura (cITP) in adult patients with an inadequate response to previous treatment [1]. Avatrombopag has achieved sustainable increases in the platelet count with an acceptable safety profile [2,3]. There are convincing data that cITP is associated with an increased risk of both arterial and venous thrombotic events which is likely multifactorial and due both to the thrombogenicity of ITP and an individual’s additional risk factors [4,5,6,7]. Based on the available literature, the risk for thrombotic complications may be higher among those patients who receive TPO-RAs [8,9,10]. However, there are no convincing prospective data available that clearly link thrombopoietin receptor agonist therapy (TPO-RA) (eltrombopag, romiplostim and avatrombopag) with venous or arterial thrombosis. 

## 2. Case #1

The first case was a 30-year-old female with a history of chronic refractory ITP that was complicated by a spontaneous intracranial bleed. She underwent decompressive hemicraniectomy and subsequently a cranioplasty. She had received multiple lines of therapy including corticosteroids, eltrombopag, azathioprine, romiplostim, and fostamatinib with romiplostim without any response. She underwent salvage splenectomy after developing intracranial hemorrhage for refractory cITP and had a good response to weekly romiplostim post-splenectomy, and was able to maintain a platelet count of >30 × 10^9^/L. She expressed an interest in changing to oral therapy and was started on avatrombopag 20 mg daily in an effort to replace the romiplostim with an oral agent.

Three weeks after starting avatrombopag and 19 months after her splenectomy, she presented to the emergency department with abdominal pain, nausea, vomiting and diarrhea. A computed tomography (CT) scan of her abdomen was unrevealing, and the patient was discharged home. Two weeks later, she presented with persistent chest and right upper quadrant abdominal pain, in addition to activity-related dizziness and lightheadedness. CT angiography of the chest revealed filling defects in the right lower lobe pulmonary arteries consistent with pulmonary embolism. On the day of her acute presentation, her platelet count was 151 × 10^9^/L. She had responded well to therapy with eltrombopag without the need for rescue medications or the need to hold doses due to elevated platelet counts. She was COVID-19-negative, but testing for a hypercoagulable state and lupus anticoagulants were not performed. Due to concerns that avatrombopag might have increased her risk for venous thromboembolism, avatrombopag was discontinued, and she was started on anticoagulation. She restarted romiplostim at her previous dose of 2 mcg/kg subcutaneously weekly for chronic ITP and has continued to do well.

## 3. Case #2

The second case was a 58-year-old female with a past medical history significant for hypothyroidism, anemia, cerebral aneurysm, peptic ulcer disease, osteoporosis and a longstanding history of cITP. She did not require therapy for ITP initially, but during follow-up developed worsening thrombocytopenia with a drop in her platelet count to below 30 × 10^9^/L and required treatment. She was treated with corticosteroids, rituximab, and IVIG without an objective response. She declined splenectomy and opted to start eltrombopag at a dose of 50 mg per day. However, she did not tolerate therapy due to side effects that included dysuria. Given this, she elected to stop eltrombopag and start avatrombopag at a dose of 20 mg daily. She had a good response to the treatment with a rising platelet count and did not require rescue medications or doses to be held due to a marked elevation of the platelet count. However, she soon developed abdominal pain and severe headaches. Given her good response, she was dose-reduced to 20 mg every other day. This ameliorated her complaints, but her platelet count dropped to less than 5 × 10^9^/L, and her dose was increased back to 20 mg daily.

She presented to the emergency department four weeks later with abdominal pain, nausea and emesis. A complete blood count demonstrated her platelet count to be normal at 150 × 10^9^/L. Testing for a lupus anticoagulant was not carried out at presentation, but 3 months later, she was found to have a lupus anticoagulant and a mildly elevated beta-2 glycoprotein 1 IgG antibody at 40.1 CU (normal 0–20). Testing for PNH was negative, inflammatory markers (C-reactive protein and sedimentation rate) were negative, and she did not have peripheral blood eosinophilia. She denied any history of peripheral arterial disease, coronary artery disease or the use of tobacco products. A CT scan of the abdomen with contrast was ordered to evaluate her abdominal pain. Imaging revealed an acute left renal vein thrombus and small areas of crescentic mural thrombus along the proximal infrarenal abdominal aorta. A CT angiogram did not show any obvious arterial injury or abnormality, and ankle–brachial index studies of both her lower extremities were negative for any signs or peripheral arterial disease.

She was started on apixaban and avatrombopag was discontinued as it was considered a possible cause of the thrombotic events. She had a platelet count of 18 × 10^9^/L at the time of her outpatient follow-up visit. She received a five-day course of prednisone, 40 mg daily, in addition to an intravenous immunoglobulin infusion (IVIG), with a suboptimal response. She was placed back on avatrombopag 20 mg daily and, eventually, it was changed to every-other-day dosing. She maintained a stable platelet count of over 30 × 10^9^/L on this dose of avatrombopag.

**Figure 1:**
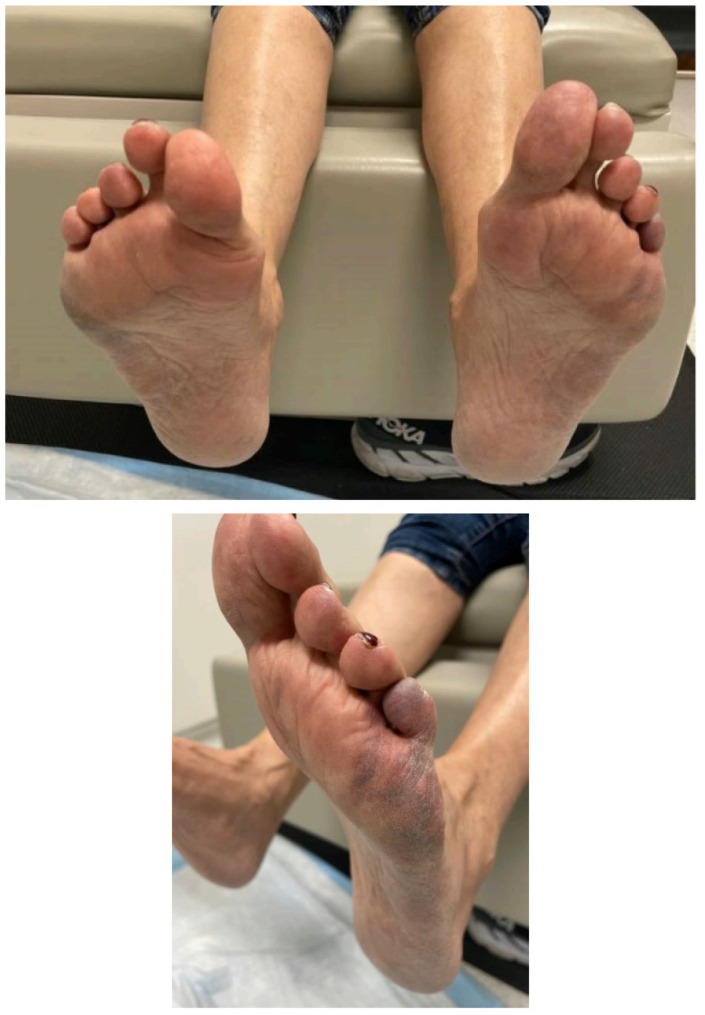
Fixed retiform purpura which was most pronounced along the distal lateral feet.

Eight weeks later after restarting the avatrombopag, she developed acute bilateral burning foot pain with attendant acral heat and erythema alternating with polar and vasospasm. Fixed retiform purpura, which was most pronounced along the distal lateral feet, rapidly evolved as well. Figure 1 demonstrates the fixed retiform purpura that was seen and more pronounced along the distal and lateral aspect of the feet. Electromyography and nerve conduction studies were normal. She was evaluated by vascular medicine and was thought to have cutaneous small vessel occlusion/in situ thrombosis with alternating erythromelalgia-like episodes and vasospasm. She had rapid improvement in her manifestations with the avatrombopag dose reduction; hence, her foot pain and acral vasomotor dysfunction were thought to be related to the avatrombopag and it was discontinued. This resulted in the complete resolution of her complaints. She was switched to romiplostim at a dose of 2 mcg/kg subcutaneously weekly and has done well since that time.

## 4. Discussion

ITP is an autoimmune disorder characterized by a combined effect of both autoantibodies against platelet membrane Gp IIb/IIIa and Gp Ib, leading to platelet destruction [10] and insufficient platelet production [11]. ITP is estimated to affect 2 to 5 persons per 100,000 [12].

There is growing evidence recognizing chronic ITP as a thrombotic disorder rather than only a hemorrhagic disorder. Based on the available epidemiological studies that compared venous/arterial thrombosis in chronic ITP patients versus the general population, the risk of VTE is increased in patients with chronic ITP and was even higher in subgroups with coexisting comorbidities, but the differences were not statistically significant [4,5,6]. The annualized incidence was 0.41–0.67 for venous thromboembolism (VTE) and 0.96–1.15 for arterial thrombosis (AT), compared to controls (0.28–0.42 and 0.67–0.91, respectively), and showed a small but statistically significantly higher risk of VTE [7]. A recent large, population-based Scandinavian cohort study that included 1821 ITP patients demonstrated an increased risk of both VTE and AT compared with the general population (IR = 16·3 per 1000 person-years, 95% confidence interval (CI): 12·8–20·6) with 39 VTE events occurring in the ITP cohort during 4233 person-years of follow-up (IR = 9·2 per 1000 person-years, 95% CI: 6·7–12·6) [4]. 

Among TPO-RAs approved for the treatment of ITP, eltrombopag and romiplostim are currently recommended as second-line agents for ITP in adults and children aged ≥1 year who are unresponsive to corticosteroids or are corticosteroid-dependent [12]. Both medications have been linked with an increased risk of thrombosis [7,8,9]. Avatrombopag is an orally administered small molecule that mimics the biological effects of thrombopoietin in vivo and in vitro [13,14]. The efficacy and safety of avatrombopag were assessed in a 6-month, multicenter, randomized, double-blind, parallel-group phase 3 study, with an open-label extension phase [2]. This study reported three thromboembolic events (one deep vein thrombosis, one pulmonary embolism, and one cerebrovascular accident), with another event of jugular vein thrombosis reported in the open-label extension phase. However, it was deemed that the study population was not large enough to assess if there was an increased risk of thromboembolic events with avatrombopag. There was a recommendation for follow-up studies to specifically evaluate the risk. In a phase II dose-finding study that assessed avatrombopag, 64 ITP patients received escalating doses of avatrombopag. Four patients (6%) developed thromboembolic complications, three of whom had risk factors for vascular disease [3].

A meta-analysis that analyzed the results of five randomized controlled trials comparing avatrombopag to placebo in patients with chronic thrombocytopenia, either secondary to chronic liver disease or primary ITP, did not show a statistically significant increase in the risk for thrombotic events with avatrombopag [15]. In a placebo-controlled study of 30 patients with thrombocytopenia secondary to chronic liver disease, avatrombopag showed an increase in absolute platelet numbers with no change in platelet reactivity found by flow cytometry [16,17].

After the development of thrombotic complications in these two reported patients, both were safely transitioned to another TPO agonist therapy after considering the available treatment options for their chronic ITP. While there was concern for the development of recurrent thrombotic events with starting another TPO agonist, the relative risk of one TPO agonist compared to another is not known. The fact that both patients had previously been treated with other TPO agonists without complications (eltrombopag and romiplostim in patient #1 and eltrombopag in patient #2) suggests that the risk for thrombotic events may not be the same for all TPO agonists. Why thrombotic complications occurred in these two patients after therapy with avatrombopag but did not occur with previous TPO agonist therapy, is not clear.

## 5. Conclusions

Avatrombopag is a promising oral therapeutic modality for chronic ITP that has demonstrated efficacy in ITP, producing a sustainable increase in the platelet count. Unlike other available TPO-RAs, avatrombopag has good oral bioavailability and does not require dietary restrictions. Despite the efficacy and ease of use, there remain questions regarding the thrombotic risks associated with the use of avatrombopag in ITP patients. A better understanding and quantification of this risk through additional studies are needed to determine if there might be subsets of ITP patients (with additional vascular risk factors) for which treatment with avatrombopag might not be safe. 

## Data Availability

Data available to share.
